# Subcutaneous emphysema and hypercarbia as a complication of laparoscopic procedure: case report

**DOI:** 10.1093/jscr/rjz415

**Published:** 2020-03-06

**Authors:** Sadal K Aldakhil, Abeer A Tashkandi, Mohammed K Al Harbi, Adel Al Shehri

**Affiliations:** 1 College of Medicine, King Saud bin Abdulaziz University for Health Sciences, Riyadh, Saudi Arabia; 2 Department of Anaesthesia, King Abdulaziz Medical City, Riyadh, Saudi Arabia

**Keywords:** subcutaneous emphysema, hypercarbia, laparoscopy

## Abstract

Subcutaneous emphysema (SE) is a rare complication of laparoscopic procedures, with an incidence rate of only 0.43–2.3%. In this report, we present a case of a 28-year-old male who underwent an elective laparoscopic inguinal hernia repair and developed surgical emphysema, hypercarbia and respiratory acidosis intraoperatively. Based on our findings, we concluded that regardless of the low incidence of SE, awareness of the associated risk factors should be ensured to avoid laparoscopic procedure-related complications.

## INTRODUCTION

Subcutaneous emphysema (SE) is defined as a “finding of gas within subcutaneous soft tissues, usually in the thorax or neck” [1]. The severity of SE has been classified according to a recent study based on the anatomical extension of SE into five grades: first including the base of the neck; second, all the neck area; third, the subpectoralis major area; fourth, the chest wall and all of the neck area and fifth, the chest wall, neck, orbit, scalp, abdominal wall, upper limbs and scrotum [2].

Previous literature from 1992 revealed that the incidence rate of SE ranges from only 0.43 to 2.3% [3]. Despite the rarity of SE, a hospital in China reported four cases of SE occurring in a one-year period during 2010 [4]. Here, we present a case of SE that developed intraoperatively as a complication of laparoscopic right inguinal hernia repair.

## CASE REPORT

A 28-year-old male, The American Society of Anesthesiologists physical status 1, was admitted for an elective laparoscopic right inguinal hernia repair with mesh. He weighed 44.4 kg and was 160 cm tall. There was no significant medical or surgical history.

Standard intraoperative monitoring was conducted including three-lead ECG and noninvasive blood pressure and end-tidal carbon dioxide (EtCO_2_) monitoring. Preoperative vital signs were SpO_2_ 100%, pulse 65/min, BP 105/67 mmHg. General and regional anesthesia was planned, and after preoxygenation, induction was initiated with 200 mg/kg propofol, 100 μg fentanyl. Endotracheal intubation was facilitated with 1 mg/kg cisatracurium and cefazolin 1 g was used as antibiotic prophylaxis. Nerve blocks with 2.5 mg/ml marcian and 8 mg dexamethasone were applied under ultrasound guidance to the transverse abdominal plane on the right and quadratus lumborum on the left.

Approximately, 30 min later, the surgery was started, and two trocars were placed, one in each side of the abdomen. A tidal volume of 400 mL at 14 breath per minute (bpm) was maintained by a ventilator. With a peak inspiratory pressure of 15 mmHg and EtCO_2_ 32 mmHg, the intra-abdominal pressure was set from 18 to 20 mmHg. After the beginning of the surgery, there was a gradual increase in the EtCO_2_, and it reached the maximum of 60 in an hour. Measures were taken to wash out CO_2_ by increasing the respiratory rate to 25 bpm, flow to 15 L/min, and FiO_2_ to 100%; however, the CO_2_ level remained high. The CO_2_ absorber was changed, along with the water trap, circuit, and filter. The temperature was normal, and the masseter tested negative in the rigidity test.

**Figure 1:**
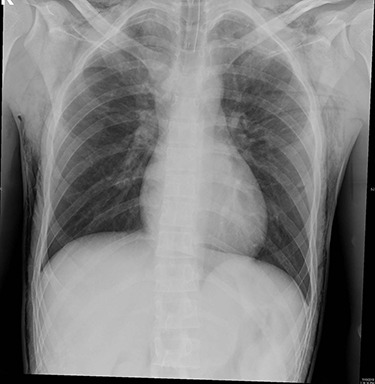
Bilateral surgical emphysema at subcutaneous tissues of the chest and root of the neck was found.

The surgeon was informed about the hypercarbia and desufflation. When examining the patient, the subcutaneous crepitus was found to be reaching up to the neck. Arterial blood gas confirmed respiratory acidosis (pH, 7.23; PaCO_2_, 56.1 mmHg; HCO_3_, 23.2 mmol/l; PaO_2_, 524 mmHg). About 15 min later, the CO_2_ level started decreasing until it reached 45 mmHg. Approximately 4 hours since the start, the operation was completed, and the patient was fully awake with stable vitals and the CO_2_ level below 45. Bedside lung ultrasound was immediately performed to exclude pneumothorax. Chest radiography was performed before extubating the patient, and bilateral surgical emphysema in the subcutaneous tissues of the chest and root of the neck was noted. Pneumothorax and pneumomediastinum were excluded (Fig. 1).

**Figure 2:**
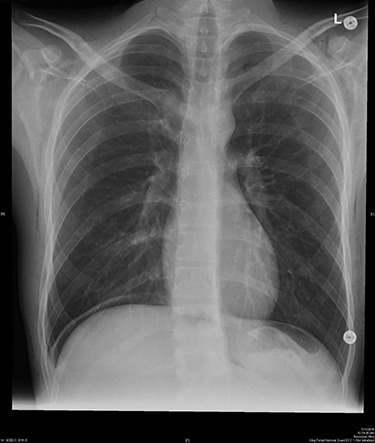
Final pre-discharged x-ray showed improvement of the SE compared to the intra-operative x-ray.

The patient was kept under observation for 24 hours. ABG was performed revealing the following values: pH 7.44; PaCO_2_ 35.9 mmHg; HCO_3_ 24.1 mmol/l and PaO_2_ 90.8 mmHg. The final pre-discharge radiography showed improvement in the SE (Fig. 2) which has been resolved spontaneously.

## Discussion

Laparoscopic procedures are overall considered safe, with a low rate of serious complications, of which up to 50% occur while gaining abdominal access or at the time of port placement [5]. While the rest of the complications occur during abdominal insufflation, tissue dissection and hemostasis [6]. The primary insufflation gas used in laparoscopic procedures to create a pneumoperitoneum is CO_2_, which has been favored over other gases due to its high solubility [7]. Some less common complications occurring during CO_2_ insufflation include pneumothorax, SE, pneumomediastinum and retroperitoneal extravasation without pneumothorax [8]. SE is also encountered as a complication of extraperitoneal and intraperitoneal laparoscopic procedures [9]. Risk factors associated with SE include the use of > 5 cannulas and procedures lasting > 3.5 hours [6]. Further, repetitive movements contribute to development of complications owing to disruption of tissue integrity and consequent structural weakness [6]. The increase in the EtCO_2_ level beyond the normal range has been frequently associated with respiratory acidosis, especially in the presence of a history of cardiovascular disease [10].

Since most such cases of complications go undetected, anesthesiologists must be vigilant and aware of the risk factors. Identification of the signs, such as sudden or persistent hypercarbia or the presence of crepitus, clinically in the early stages of SE development may help prevent and manage such cases more effectively. SE tends to resolve spontaneously in most cases; because of the high diffusion rate of CO_2_, it takes up to 24 hours for SE to resolve [11]. However, the patient should be monitored closely [12]. The mainstay treatment for SE is peritoneal desufflation. Further, intraoperative chest radiography and arterial blood gas analysis can be employed to exclude other differentials. In cases of severe SE, surgical intervention, such as drainage and incision, may sometimes be needed to decrease the pressure [11].

The risk factors in our case included an intra-abdominal pressure exceeding 15 mmHg, end tidal CO_2_ > 50 mmHg, and the long duration of the procedure. However, most of the symptoms resolved after 24 hours, and the subcutaneous crepitus level reached T4. The postoperative management employed in this case was majorly supportive, with only radiography performed to monitor the condition.

In conclusion, regardless of the low incidence of SE, awareness of the associated risk factors should be ensured to avoid laparoscopic procedure-related complications.
